# Supplementary material for: Sustained-input switches for transcription factors and microRNAs are central building blocks of eukaryotic gene circuits
Source: Genome Biol. 2013 Aug 23;14(8):R85. doi: 10.1186/gb-2013-14-8-r85 (PMC4054853; doi:10.1186/gb-2013-14-8-r85)
Supplement: Additional file 5 — HTML Browsable Motif Output. Zipped folder containing all WaRSwap and FANMOD motif output, viewable in a web browser. [file gb-2013-14-8-r85-S5.ZIP › HTML_browsable_motif_output/FANMOD_ath_tair9/sigs_fanmodm-2000.pvals.heatmaps.html/motif_id_6_000000110_tftype_ath_upstream_-2000_0.html]

```
BG_MODEL = FANMOD
MOTIF_ID = 6_000000110
TF_TYPE = ath
UPSTREAM = -2000_0


PVals
FN_0.2	FN_0.4	FN_0.6	FN_0.8
dg_60.genes	0.326	0.989	0.98	0
dg_70.genes	0.33	0.987	0.983	0
dg_80.genes	0.344	0.993	0.986	0

ZScores
FN_0.2	FN_0.4	FN_0.6	FN_0.8
dg_60.genes	0.428	-2.254	-2.057	2.747
dg_70.genes	0.435	-2.264	-2.065	2.673
dg_80.genes	0.381	-2.329	-2	2.784

StDevs
FN_0.2	FN_0.4	FN_0.6	FN_0.8
dg_60.genes	33.566	39.161	25.54	6.006
dg_70.genes	33.083	39.98	25.274	6.143
dg_80.genes	34.211	38.305	25.704	5.91
```
